# *Ganoderma lucidum* polysaccharide ameliorates cholesterol gallstone formation by modulating cholesterol and bile acid metabolism in an FXR-dependent manner

**DOI:** 10.1186/s13020-024-00889-y

**Published:** 2024-01-24

**Authors:** Dan Huang, Shuang Shen, Qian Zhuang, Xin Ye, Yueqin Qian, Zhixia Dong, Xinjian Wan

**Affiliations:** Digestive Endoscopic Center, Shanghai Sixth People’s Hospital Affiliated to Shanghai Jiao Tong University School of Medicine, No. 600 Yishan Road, Shanghai, 200233 China

**Keywords:** Cholesterol gallstones, *Ganoderma lucidum* polysaccharide, Farnesoid X receptor, Cholesterol, Bile acids, Intestinal flora

## Abstract

**Background:**

Cholesterol gallstone (CG) disease is a worldwide common disease characterized by cholesterol supersaturation in gallbladder bile. *Ganoderma lucidum* polysaccharide (GLP) has been shown to possess various beneficial effects against metabolic disorders. However, the role and underlying mechanism of GLP in CG formation are still unknown. This study aimed to determine the role of GLP in ameliorating lithogenic diet (LD)-induced CG formation.

**Methods:**

Mice were fed either a normal chow diet, a LD, or LD supplemented with GLP. Real-time quantitative polymerase chain reaction (RT-qPCR) and western blotting were used to detect the expression of genes involved in cholesterol and bile acid (BA) metabolism. The BA concentrations in the ileum were quantified by liquid chromatography-tandem mass spectrometry (LC–MS/MS). The microbiota in cecal contents were characterized using 16S ribosomal RNA (16S rRNA) gene sequencing.

**Results:**

GLP effectively alleviated CG formation induced by LD. Specifically, GLP reduced the total cholesterol (TC) levels, increased the total BA levels, and decreased the cholesterol saturation index (CSI) in gallbladder bile. The protective effect of GLP was attributed to the inhibition of farnesoid X receptor (FXR) signaling, increased hepatic BA synthesis and decreased hepatic cholesterol synthesis and secretion. GLP also altered the BA composition in the ileum, reducing FXR-agonistic BAs and increasing FXR-antagonistic BAs, which may contribute to the inhibition of intestinal FXR signaling. Additionally, GLP improved dysbiosis of the intestinal flora and reduced the serum levels of hydrogen sulfide (H_2_S), a bacterial metabolite that can induce hepatic FXR, thereby inhibiting hepatic FXR signaling. Moreover, the protective effect of GLP against CG formation could be reversed by both the global and gut-restricted FXR agonists.

**Conclusions:**

Taken together, GLP ameliorates CG formation by regulating cholesterol and BA metabolism in an FXR-dependent manner. Our study demonstrates that GLP may be a potential strategy for the prevention against CG disease.

**Supplementary Information:**

The online version contains supplementary material available at 10.1186/s13020-024-00889-y.

## Background

Cholelithiasis is a common and frequently-occurring disease worldwide. Cholesterol gallstones (CGs) account for almost 90% of the gallstones found during cholecystectomy [1]. Cholecystectomy is the definitive treatment but leads to complications that seriously threaten patient’s health and quality of life [2]. Ursodeoxycholic acid (UDCA) is a first-line drug for the treatment of CGs in the gallbladder, but it also presents the disadvantages of long treatment duration and high recurrence rate [3]. Therefore, more attention should be paid to the prevention of CG disease, and it is necessary to look for new methods to prevent CG disease.

Supersaturation of biliary cholesterol is a prerequisite for CG formation [4]. Excess cholesterol and/or inadequate bile acids (BAs) or phospholipids (PLs) contribute to the supersaturation of bile. Cholesterol biosynthesis is regulated through SREBP cleavage-activating protein/sterol regulatory element-binding protein 2 (SCAP/SREBP2) pathway [5, 6]. ATP-binding cassette subfamily G members 5 and 8 (ABCG5/G8) mediate cholesterol secretion in liver [7]. The synthesis of BAs is the primary route for eliminating excess cholesterol [8]. In the liver, BAs are synthesized from cholesterol via cholesterol 7α-hydroxylase (CYP7A1), sterol 12α-hydroxylase (CYP8B1), sterol 27-hydroxylase (CYP27A1) and oxysterol 7α-hydroxylase (CYP7B1) [9]. BAs are secreted into the capillary bile duct of hepatocytes by bile salt export pump (BSEP) and multidrug resistance-associated protein 2 (MRP2) [10]. Approximately 95% of the BAs are reabsorbed from the terminal ileum and returned to the liver. PLs are transported into bile by ATP-binding cassette subfamily B member 4 (ABCB4) [11].

As a BA sensor, the farnesoid X receptor (FXR), a nuclear receptor mainly expressed in the intestine and liver, plays a crucial role in regulating cholesterol and BA metabolism [12]. Activation of FXR is the primary mechanism in inhibiting BA synthesis by directly inducing target genes, including intestinal fibroblast growth factor 15 (FGF15) and hepatic small heterodimer partner (SHP), which in turn reduces the expression of genes encoding BA synthetic enzymes [13]. BAs are the endogenous ligands of FXR. Cholic acid (CA) and deoxycholic acid (DCA) are FXR agonists, while β-muricholic acid (β-MCA) is a FXR antagonist [14, 15]. Furthermore, the bacterial metabolite hydrogen sulfide (H_2_S) is shown to activate hepatic FXR [16].

*Ganoderma lucidum* is a traditional Chinese medicine and has been reported to possess various pharmacological effects such as anticancer, hypoglycemic, immunomodulatory and antioxidant effects [17–19]. *Ganoderma lucidum* polysaccharide (GLP) is a water-soluble polysaccharide and one of the main bioactive components extracted from *Ganoderma lucidum*. Several studies reported that GLP could modulate the gut microbiota composition, which might be related to the prevention and treatment of diabetes and obesity [18, 20]. However, it is unclear whether GLP can prevent CG formation.

This study aimed to explore the impact and potential mechanism of GLP on lithogenic diet (LD)-induced CG formation. Our findings indicated that GLP effectively attenuated CG formation and cholesterol supersaturation of gallbladder bile in mice. We found that GLP decreased the levels of ileal CA and DCA, increased the levels of ileal β-MCA, ameliorated gut dysbiosis, and lowered serum H_2_S levels, which collectively inhibited LD-induced activation of intestinal and hepatic FXR signaling, thereby promoting hepatic BA synthesis and increasing cholesterol depletion. Notably, GLP also suppressed hepatic cholesterol synthesis and secretion. The protective effect of GLP against CG formation was reversed by both global and gut-restricted FXR agonists. Our study may provide a scientific basis for using GLP as a promising strategy for preventing CG disease.

## Methods

### Preparation and characterization of GLP

GLP was provided by Ci Yuan Biotechnology Co., Ltd. (Shanxi, China). Briefly, GLP was extracted and purified through hot water extraction, centrifugation, concentration, precipitation, deproteinization, dialysis and lyophilization as previously described [21]. The molecular weight of GLP was measured by high-performance gel permeation chromatography (HPGPC) according to the previous method [22]. The monosaccharide composition of GLP was analyzed by high-performance liquid chromatography (HPLC) as described before [23]. Attenuated total reflectance Fourier transform infrared (ATR-FTIR) analysis was carried out to identify the functional groups in GLP as described previously [24]. In our study, the weight-average molecular weight of GLP was 42.3 kDa (Additional file 1: Fig. S1), the major monosaccharides of which were glucose (66.67%), galactose (8.59%), mannose (8.26%), glucosamine (7.57%) and glucuronic acid (3.82%) (Additional file 1: Fig. S2A, B). In addition, the ATR-FTIR spectrum showed that GLP displayed the typical absorption bands for polysaccharides centered at 3244 cm^−1^ (hydroxyl O–H stretching vibration), 2935 cm^−1^ (methylene C–H stretching vibration), 2881 cm^−1^ (methyl C–H stretching vibration), 1387 cm^−1^ (hydroxyl O–H bending vibration) and 1203 cm^−1^ (C–O stretching vibration) (Additional file 1: Fig. S3).

### Animal study

All animal experiments were approved by the Institutional Animal Care and Use Committee of Shanghai Sixth People's Hospital Affiliated to Shanghai Jiao Tong University School of Medicine (approval number: 2021-0063). C57BL/6J mice (male, 6 weeks old) were purchased from Shanghai SLAC Laboratory Animal Co., Ltd. (Shanghai, China). All the mice were maintained under 12-h light/dark cycles at 20–22 °C and 45 ± 5% humidity, and had access to food and water ad libitum. Mice were allowed to acclimate for 1 week before experiments. The normal chow diet (LAD 0011) and LD (TP 28900) were both purchased from Trophic Diet (Nantong, China). The LD contained 15% fat, 1.25% cholesterol, and 0.5% CA. GLP was purchased from Ci Yuan Biotechnology Co., Ltd. (Shanxi, China) and the purity of polysaccharide from *Ganoderma lucidum* was 98%. The LD pellets were first broken to the powder form, next, GLP was added in the LD powder to the finial concentration of 2% or 4%, and then mixed well and reshaped them from powder to pellets in the sterile condition. Fexaramine was purchased from Biochempartner Co., Ltd. (Shanghai, China) and GW4064 was purchased from Selleck (Houston, TX, USA).

In the GLP intervention study, 40 mice were randomly divided into 4 groups (n = 10 per group and n = 5 per cage) and fed with (i) a normal chow diet (ND group); (ii) a lithogenic diet (LD group); (iii) a LD supplemented with low-dose GLP (2%; GLPL group); and (iv) a LD supplemented with high-dose GLP (4%; GLPH group), all for 6 weeks. The body weights of mice were recorded once a week.

For the FXR regulation study, 40 mice were randomly divided into 4 groups (n = 10 per group and n = 5 per cage), (i) vehicle fed LD (LD group), (ii) LD supplemented with 4% GLP (LD + GLP group), and (iii) supplemented with 4% GLP coupled with 100 mg kg^−1^ d^−1^ Fexaramine by gavage (LD + GLP + Fexaramine group) or 100 mg kg^−1^ d^−1^ GW4064 by gavage (LD + GLP + GW4064 group), all for 6 weeks. The body weights of mice were recorded once a week.

At the end of experiments, mice were fasted overnight before being euthanized. Blood samples were collected and centrifuged to obtain the serum sample. Tissues including gallbladder, liver, ileum and cecum contents were obtained and then stored at − 80 °C for further analysis.

### Analyses of biliary, serum and hepatic lipids and calculation of cholesterol saturation index (CSI)

The levels of total cholesterol (TC) and total BAs in gallbladder bile and levels of TC, total triglyceride (TG) and total BAs in serum were measured using commercially available kits (Jiancheng Bioengineering Institute, Nanjing, China). The level of total PLs in gallbladder bile was measured according to the manufacturer’s instruction of the kit (Wako Pure Chemicals, Osaka, Japan). The CSI of gallbladder bile was calculated according to Carey’s critical table [25]. Hepatic lipids were extracted by the Folch method as previously described [26]. The hepatic levels of TC, TG and total BAs were measured using kits from Jiancheng Bioengineering Institute, as described.

### Histology of liver tissue

The liver tissues were fixed in 4% paraformaldehyde, embedded in paraffin, cut into 4-µm slices and then stained with hematoxylin and eosin (H&E) for histological analysis. Hepatic lipid accumulation was measured by Oil Red O staining as previously described [27]. The positively stained area was quantified by ImageJ software and results were expressed as a percentage of the total area of a high-power field.

### RNA extraction and real-time quantitative polymerase chain reaction (RT-qPCR)

Total RNA in liver and ileum tissues was extracted using TRIzol (Invitrogen, CA, USA). The quantity and purity of RNA were determined by a spectrophotometer (Thermo Fisher Scientific, Waltham, MA, USA). Total RNA was reverse transcribed using the PrimeScript™ RT reagent kit (Takara Biotechnology, Japan), and then the SYBR® Premix Ex Taq™ reagent kit (Takara Biotechnology, Japan) was used to perform qPCR. The mRNA levels of target genes were normalized to glyceraldehyde 3-phosphate dehydrogenase (GAPDH) using the 2^−ΔΔCt^ method and the relative expression levels were shown as fold changes relative to the control group. The sequences of primers are listed in Table 1.

**Table 1:** Primers for qPCR

Gene	Primer sequence (5′ to 3′)	
FXR	Forward	GGGATGAGCTGTGTGTTGTCTGTG
	Reverse	GTCTGTTGGTCTGCCGTGAGTTC
FGF15	Forward	CGGCAAGATATACGGGCTGATTCG
	Reverse	TCTACATCCTCCACCATCCTGAACG
FGFR4	Forward	GGTGACCGAGGATGATGTGATGAAG
	Reverse	AGAGACAGCCAGCAGGACCTTG
SHP	Forward	CAGGTCGTCCGACTATTCTGTATGC
	Reverse	TACCGCTGCTGGCTTCCTCTAG
CYP7A1	Forward	GTATGCCTTCTGCTACCGAGTGATG
	Reverse	GTGCGTCTTAGCCTTCTCCATGTC
CYP8B1	Forward	TGGACAGCGTGATGGAGGAGAG
	Reverse	GGTGGATCTTCTTGCCCGACTTG
CYP27A1	Forward	AAGGACCACCGAGACCACAAGG
	Reverse	GGAGGGTTTCAGGCAGCCAATC
CYP7B1	Forward	TTGGCTTCCTTATCTTGGCATGGC
	Reverse	TCGCTGATAATCGGCTGCTGAAC
BSEP	Forward	GAGTTGGTGTGGCTGTCCTTATCC
	Reverse	ATCTGGTCGGCAATGGCTTCATC
MRP2	Forward	CCTATTCCTGCCTGTTCTTCGTCTC
	Reverse	TCTGCTTCTTGGTCAATCCGTGTG
SCAP	Forward	TACAGCAGCAGCAACACAGTGAC
	Reverse	ACAGGAAGCGGTACAGGTGAGG
SREBP2	Forward	CAAAGAAGGAGAGAGGCGGACAAC
	Reverse	GGCGGAGACATCAGAAGGACATTC
ABCG5	Forward	CATCGCAGCCCAACTCCTTCAG
	Reverse	CCACAGAACACCAACTCTCCGTAAG
ABCG8	Forward	GCTCGTGTGGTTGGTGGTCTTC
	Reverse	GTTGCCGATTTGTGTGGTGTAAAGG
GAPDH	Forward	AGG​TCG​GTG​TGA​ACG​GAT​TTG
	Reverse	TGT​AGA​CCA​TGT​AGT​TGA​GGT​CA

### Western blot analysis

Ileum and liver tissues were homogenized in RIPA lysis buffer containing protease and phosphatase inhibitors (Beyotime Institute of Biotechnology, Shanghai, China). Equal amounts of proteins were separated by SDS-PAGE and then transferred to polyvinylidene difluoride membranes (Millipore, Tullagreen, Ireland). The membranes were blocked, and then incubated with primary antibodies against FGF15 (1:800, sc-398338; Santa Cruz Biotechnology, USA), SHP (1:800, A1836; ABclonal Technology, Wuhan, China), CYP7A1 (1:1000, ab234982; Abcam, Cambridge, UK), CYP7B1 (1:1000, 24889-1-AP; Proteintech, Rosemont, IL, USA), SREBP2 (1:1000, ab30682; Abcam, Cambridge, UK), ABCG5 (1:1000, 27722-1-AP; Proteintech, Rosemont, IL, USA), ABCG8 (1: 1000, DF6673; Affinity Biosciences, Changzhou, China), and GAPDH (1:5000, 60004-1-Ig; Proteintech, Rosemont, IL, USA) at 4 °C overnight, followed by incubation with horseradish peroxidase conjugated secondary antibodies. The blots were developed using the enhanced chemiluminescence (ECL) method. The gray values of the bands were calculated using ImageJ software and were normalized to GAPDH. The relative expression levels were shown as fold changes relative to control group.

### BA analysis

BA concentrations in the ileum tissues were measured using negative electrospray liquid chromatography-tandem mass spectrometry (LC–MS/MS). The 47 kinds of BA standards were weighed accurately and prepared as standard solutions through serial dilution using methanol. The ileum samples were weighed precisely (25 ± 5 mg), homogenized in 50 μL of internal standard working solution and 350 μL of extraction mixture (methanol/water, 4:1), and centrifuged at 13,000×*g* at 4 °C for 15 min. The supernatant was dried with nitrogen, re-dissolved with 100 μL of 50% acetonitrile, vortex-mixed for 30 s, and centrifuged at 13,000×*g* at 4 °C for 15 min. The supernatant was then ready for LC–MS/MS. Chromatographic separation was achieved on an Acquity ultraperformance liquid chromatography (UPLC) BEH C18 column (internal dimensions: 150 mm × 2.1 mm, 1.7 µm; Waters). The temperature of the column was set to 50 °C. The injection volume was set at 5 μL. The mobile phase consisted of 0.025% formic acid and 10 mM ammonium acetate in water (solvent A), and acetonitrile/methanol (9:1) solution (solvent B). The flow rate was set at 0.4 mL/min. The following gradient pattern was used for the separation of BAs: 0–0.5 min, 5–10% B; 0.5–1.0 min, 10–20% B; 1.0–2.0 min, 20–28% B; 2.0–8.0 min, hold at 28% B; 8.0–11.0 min, 28–33% B; 11.0–20.0 min, 33–45% B; 20.0–23.0 min, 45–50% B; 23.0–29.0 min, 50–80% B; 29.0–29.01 min, 80–100% B; 29.01–31.0 min, hold at 100% B; 31.0–31.01 min, 100–5% B; and 31.01–33.0 min, hold at 5% B. Analytes were detected by electrospray ionization and quantified by internal standard methods.

### 16S ribosomal RNA (16S rRNA) gene sequencing

Total bacterial genomic DNA from cecal contents was extracted using QIAamp DNA Stool Kit (Qiagen, Germany) according to the manufacturer’s guidelines. The V3-V4 regions of the bacterial 16S rRNA genes were amplified with specific primers by PCR. Amplicons were extracted from 2% agarose gel, purified with the AxyPrep DNA Gel Extraction Kit (Axygen Biosciences, Union City, CA, USA), then quantified by QuantiFluor™-ST (Promega, USA) and sequenced on the Illumina MiSeq platform (Illumina, San Diego, USA).

The raw Illumina fasta files were demultiplexed, quality filtered, and analyzed using the Quantitative Insights into Microbial Ecology (QIIME, v1.9.1) platform with the following criteria: (i) reads were truncated at any site receiving a quality score of < 20, with the truncated reads shorter than 50 bp being discarded; (ii) only sequences that overlap longer than 10 bp were assembled according to their overlapped sequence, and reads that could not be assembled were discarded; and (iii) exact barcode matching, with 2 nucleotide mismatches in primer matching. Sequences with ≥ 97% similarity were assigned to the same operational taxonomic units (OTUs) according to sequences analysis using UPARSE v7.1 [28]. The taxonomy of each OTU representative sequence was analyzed using the Ribosomal Database Project (RDP) Classifier v2.2 against the 16S rRNA gene database using a confidence threshold of 0.7 [29]. Alpha diversity index including Ace, Chao, Shannon, Sobs, and beta diversity index (principal coordinate analysis (PCoA)) were calculated with QIIME.

### Serum H_2_S and FGF15 detection

The level of serum H_2_S was determined by spectrophotometry as previously described using NaHS (2.5–200 μM) as a standard [30]. Briefly, 75 μL of serum was mixed with 250 μL of 1% zinc acetate, 425  μL distilled water, 20 mM *N*,*N*-dimethyl-*p*-phenylenediamine sulfate in 7.2 mM HCl (133 μL) and 30 mM FeCl_3_ in 1.2 mM HCl (133 μL). Protein was precipitated by adding 250 μL of 10% trichloroacetic acid. After centrifugation, the absorbance of the supernatant was measured with a spectrophotometer at 670 nm.

The level of serum FGF15 was measured using a sandwich ELISA Kit (Bioswamp, Wuhan, China) according to the manufacturer’s instruction.

### Statistical analysis

Statistical analysis was undertaken using GraphPad Prism v7.0 (La Jolla, CA, USA). All data were presented as the mean ± standard error of the mean (SEM). For data that showed a normal distribution, significant differences were identified by one-way analysis of variance (ANOVA) for multiple comparisons. For data with a skewed distribution, significant differences were identified by Kruskal–Wallis test for multiple comparisons. Statistical significance was indicated by p values < 0.05. All the bar plots were generated with GraphPad Prism v7.0.

## Results

### GLP attenuated CG formation and metabolic disorders in LD-fed mice

**Fig. 1:**
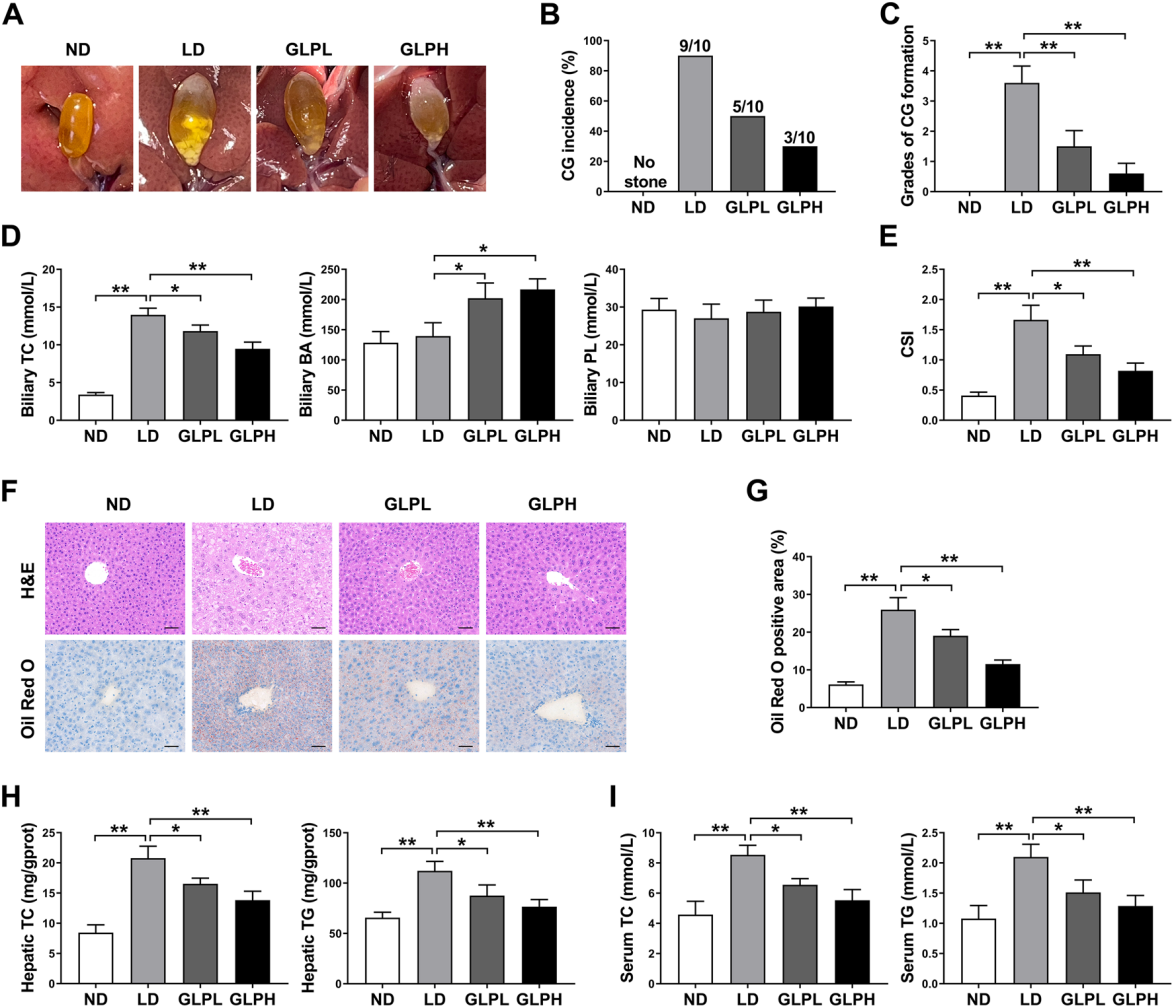
GLP ameliorated LD-induced CG formation and metabolic disorders. **A** Representative gallbladders and gallstones. **B** Incidence of gallstone. **C** Grades of experimental CGs. **D** Concentrations of TC, BA and PL in gallbladder bile. **E** CSI of gallbladder bile. **F** Representative images of H&E and Oil Red O staining of liver tissue sections. Scale bars, 50 μm. **G** Percentage of Oil Red O positive area. **H** Hepatic TC and TG levels. **I** Serum TC and TG levels. Data are shown as the mean ± SEM (n = 10). *p < 0.05, **p < 0.01. ND: normal diet; LD: lithogenic diet; GLPL: low-dose *Ganoderma lucidum* polysaccharide; GLPH: high-dose *Ganoderma lucidum* polysaccharide; CG: cholesterol gallstone; TC: total cholesterol; BA: bile acid; PL: phospholipid; CSI: cholesterol saturation index; H&E: hematoxylin and eosin; TG: triglyceride

LD-induced CG model in mice was used to investigate the impact of GLP on CG formation. The gallbladders of mice in the ND group did not have CG formation and were filled with clear bile. In contrast to the ND group, CGs formed in 90% (9/10) of the mice in the LD group. With GLP supplementation, the incidence of CG formation was significantly reduced to 50% (5/10) and 30% (3/10) in the GLPL group and GLPH group respectively (Fig. 1A, B). The grading criteria described previously was adopted to grade CGs [31]. As shown in Fig. 1C, GLP remarkably decreased the grade of CGs in LD-fed mice in a dose-related manner. There were no obvious differences in body weight gain among the four groups (Additional file 1: Fig. S4A). We further analyzed lipid composition in gallbladder bile. Compared with the ND group, biliary TC level and CSI were increased markedly in the LD group, whereas GLP supplementation noticeably reduced biliary TC level and CSI in a dose-dependent manner. GLP also significantly increased total biliary BA level. However, total biliary PL level was not changed significantly by GLP supplementation (Fig. 1D, E and Additional file 1: Fig. S4B). These results demonstrated that GLP could attenuate LD-induced CG formation and biliary cholesterol supersaturation.

Metabolic syndrome (MS) has been demonstrated to be associated with the incidence of gallstones [32]. As shown in Fig. 1F, G, LD-fed mice exhibited obvious accumulation of lipid droplets in the liver, whereas GLP alleviated hepatic steatosis and lipid deposition dose-dependently. Furthermore, GLP significantly decreased TC and TG levels both in the liver and serum, which were increased by LD consumption (Fig. 1H, I). These data indicated that GLP supplementation could improve LD-induced metabolic disorders.

### GLP inhibited intestinal FXR-FGF15 and hepatic FXR-SHP signaling

**Fig. 2:**
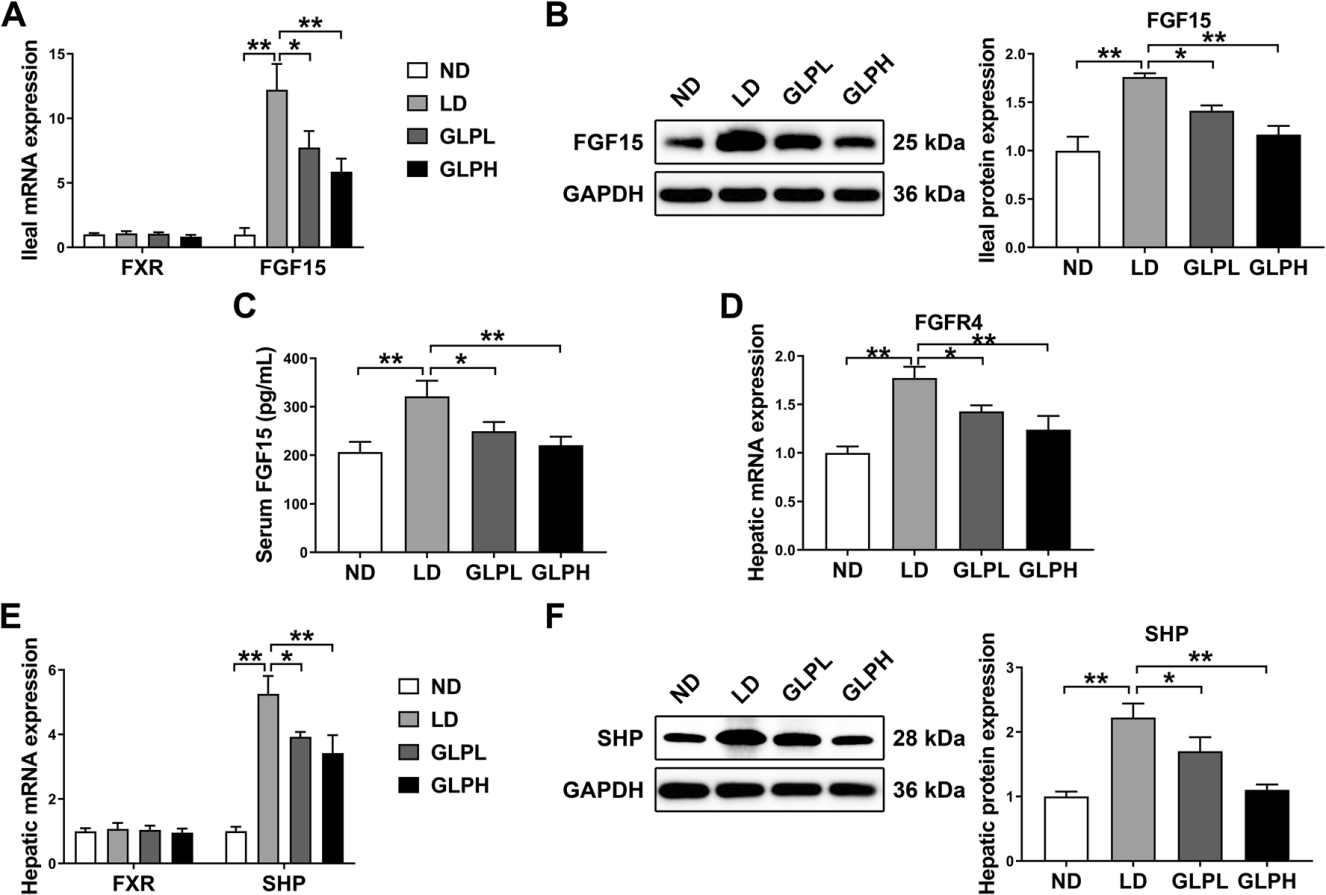
GLP downregulated intestinal FXR-FGF15 and hepatic FXR-SHP signaling. **A** The mRNA expression of FXR and FGF15 in the ileum (n = 8). **B** The protein expression and quantification of FGF15 in the ileum (n = 6). **C** Serum FGF15 level (n = 10). **D**, **E** The mRNA expression of FGFR4, FXR and SHP in the liver (n = 8). **F** The protein expression and quantification of SHP in the liver (n = 6). The mRNA expression and protein expression were normalized to GAPDH and shown as fold changes relative to control group. Data are shown as the mean ± SEM. *p < 0.05, **p < 0.01. ND: normal diet; LD: lithogenic diet; GLPL: low-dose *Ganoderma lucidum* polysaccharide; GLPH, high-dose *Ganoderma lucidum* polysaccharide

It is widely known that the FXR signaling pathway plays a crucial role in regulating hepatic metabolism and is closely associated with CG formation. Thus, to further determine whether this pathway is involved in the beneficial effect of GLP on CG formation, FXR and its downstream molecules in both ileum and liver were detected. As shown in Fig. 2A, B, the expression of FGF15 was found to be increased in distal ileum of LD-fed mice at both the mRNA and protein levels, while GLP supplementation significantly decreased both the mRNA and protein levels of FGF15. Consistent with these findings, serum FGF15 level was higher in the LD group compared to the ND group, while GLP supplementation effectively reversed the upregulation (Fig. 2C).

Intestinal-derived FGF15 is secreted into the portal vein and circulates to the liver, where it binds to fibroblast growth factor receptor 4 (FGFR4) and acts to inhibit the synthesis of BAs from cholesterol [13]. As shown in Fig. 2D, hepatic FGFR4 mRNA expression was increased in parallel with the increased ileal FGF15 mRNA and protein expression and serum FGF15 levels in LD-fed mice, while this upregulation was effectively reversed upon GLP supplementation. Additionally, both the mRNA and protein levels of SHP in the liver were significantly elevated in the LD group compared to the ND group, while GLP supplementation dramatically reduced its expression (Fig. 2E, F).

Taken together, these data suggested that GLP supplementation downregulated FXR-FGF15 signaling pathway in the intestine and FXR-SHP signaling pathway in the liver, both of which were activated by LD consumption.

### GLP modulated synthesis and transport of BAs and cholesterol

**Fig. 3:**
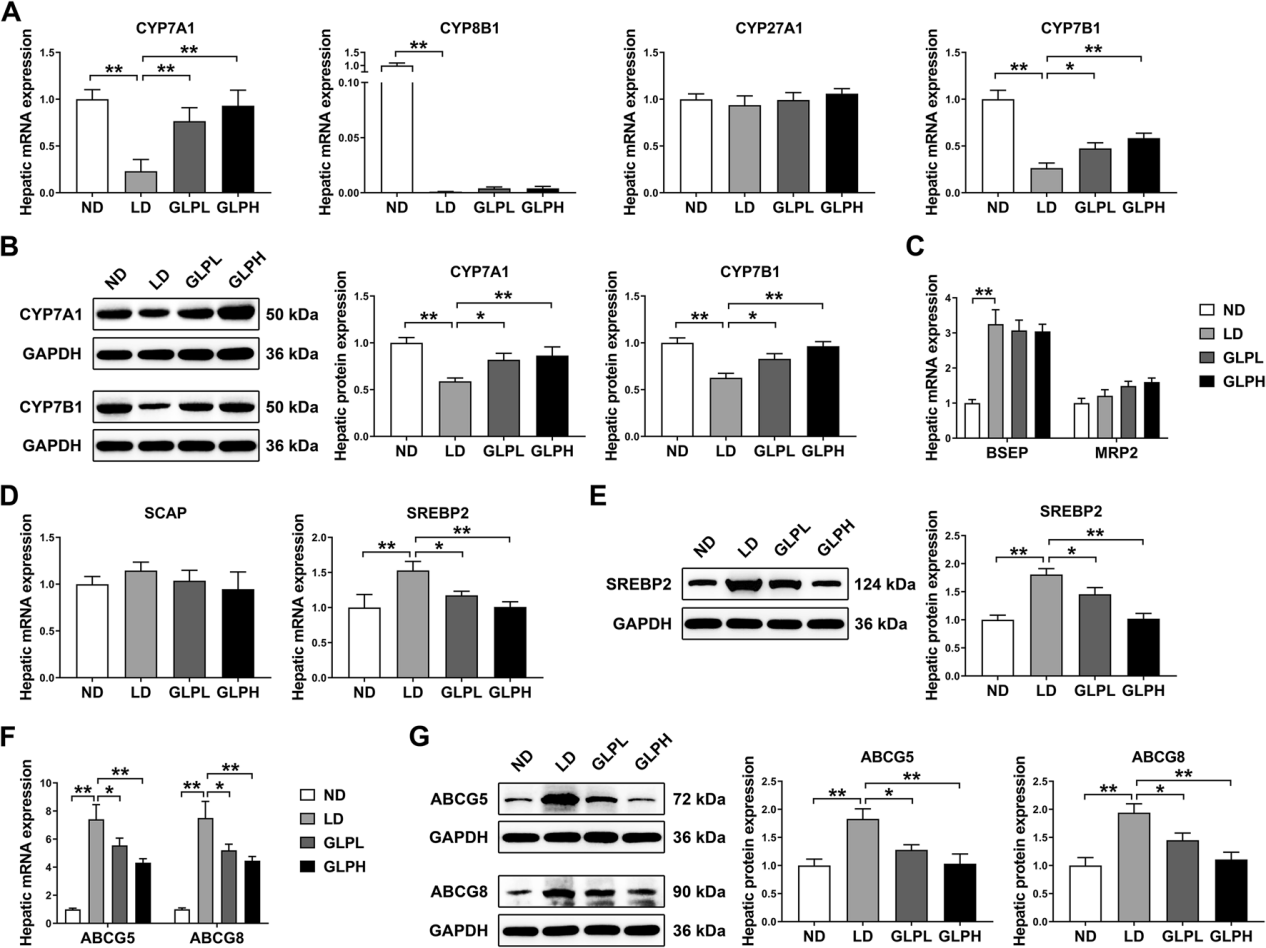
GLP promoted BA synthesis and inhibited cholesterol synthesis and efflux in liver. **A** The mRNA expression of CYP7A1, CYP8B1, CYP27A1 and CYP7B1 in the liver (n = 8). **B** The protein expression and quantification of CYP7A1 and CYP7B1 in the liver (n = 6). **C**, **D** The mRNA expression of BSEP, MRP2, SCAP and SREBP2 in the liver (n = 8). **E** The protein expression and quantification of SREBP2 in the liver (n = 6). **F** The mRNA expression of ABCG5 and ABCG8 in the liver (n = 8). **G** The protein expression and quantification of ABCG5 and ABCG8 in the liver (n = 6). The mRNA expression and protein expression were normalized to GAPDH and shown as fold changes relative to control group. Data are shown as the mean ± SEM. *p < 0.05, **p < 0.01. ND: normal diet; LD: lithogenic diet; GLPL: low-dose *Ganoderma lucidum* polysaccharide; GLPH: high-dose *Ganoderma lucidum* polysaccharide; BA: bile acid

Consistent with the activation of FXR induced by LD, we found that hepatic mRNA expression of CYP7A1, CYP8B1 and CYP7B1 were significantly decreased by LD consumption compared to the ND group. However, GLP supplementation significantly upregulated the mRNA expression of CYP7A1 and CYP7B1 but did not significantly affect the mRNA expression of CYP8B1. There was no significant difference in hepatic CYP27A1 mRNA expression among groups (Fig. 3A). Moreover, western blotting analysis confirmed that GLP supplementation effectively reversed the LD-induced reduction of hepatic CYP7A1 and CYP7B1 protein expression (Fig. 3B). Consistent with the increased BA biosynthesis from cholesterol, hepatic TC level was reduced by GLP supplementation (Fig. 1H). BSEP and MRP2 are hepatic BA efflux transporters. We observed that mice fed the LD showed higher BSEP mRNA levels compared to ND-fed mice, but GLP did not significantly alter the mRNA expression of BSEP in the liver. Hepatic MRP2 mRNA expression remained unchanged among groups (Fig. 3C). Moreover, hepatic mRNA expression of ABCB4, a major phospholipid transporter, did not show significant differences among groups (Additional file 1: Fig. S5).

To further explore the role of cholesterol metabolism in liver, we detected the expression of genes involved in hepatic cholesterol synthesis and efflux. SREBP2 and SCAP are key regulators in cholesterol synthesis. GLP supplementation dramatically downregulated LD-induced mRNA expression of SREBP2 in the liver. However, hepatic SCAP mRNA expression was not significantly affected by GLP (Fig. 3D). Western blotting analysis further confirmed that GLP significantly inhibited LD-induced SREBP2 protein expression in liver (Fig. 3E). Moreover, both the mRNA and protein levels of ABCG5 and ABCG8, important cholesterol efflux transporters, were dramatically increased in the LD group compared to the ND group, while GLP significantly reversed the expression of these genes (Fig. 3F, G). These results were consistent with the reduction of cholesterol level in gallbladder bile by GLP supplementation (Fig. 1D and Additional file 1: Fig. S4B).

Taken together, these data indicated that GLP promoted BA synthesis while inhibiting cholesterol synthesis and efflux, which may contribute to the reduction of CSI in bile.

### GLP reduced FXR-agonistic BAs and increased FXR-antagonistic BAs in ileum

**Fig. 4:**
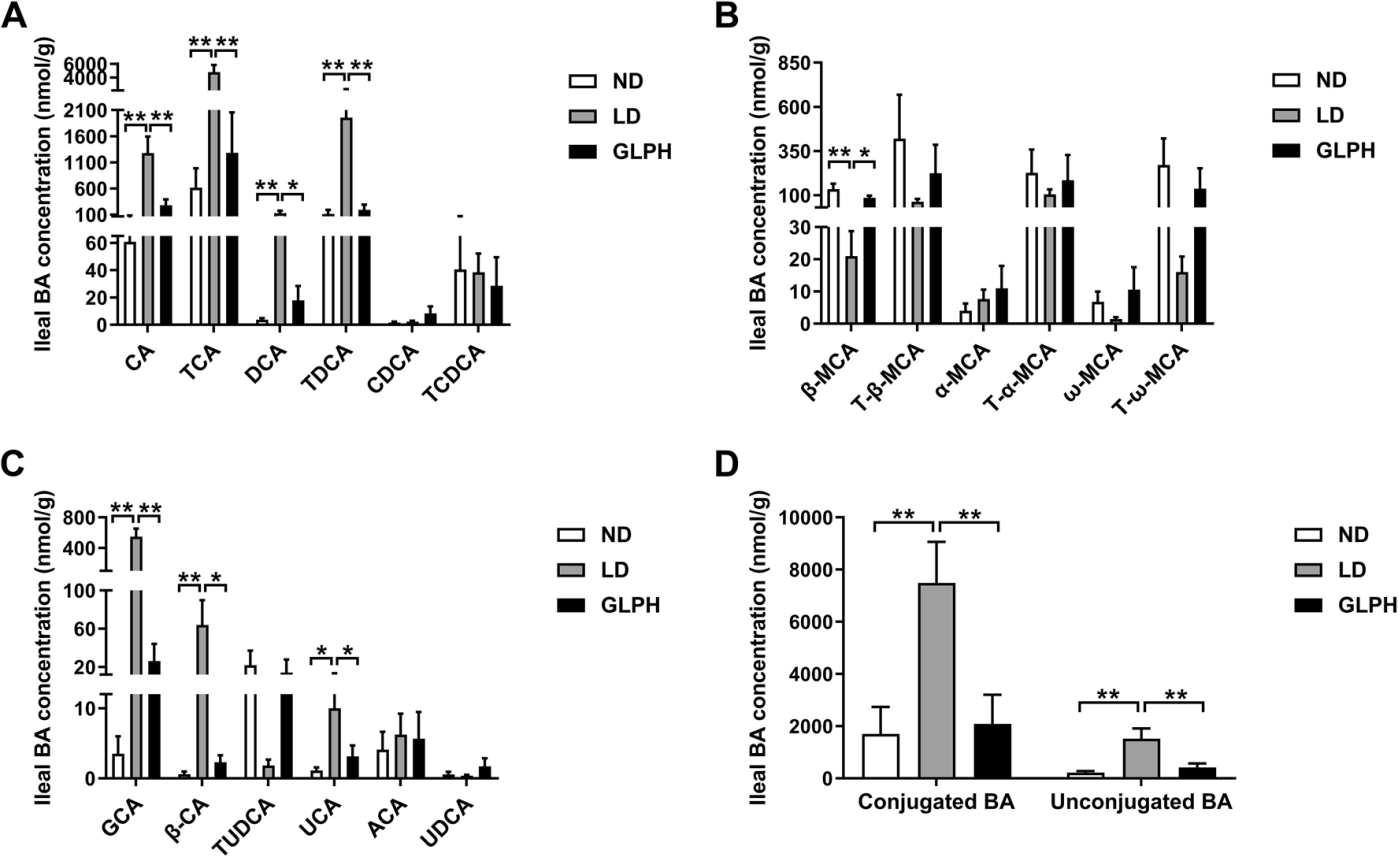
GLP changed the BA composition in the ileum. **A–C** The top 18 most abundant ileal BA species are shown. **D** Concentrations of total conjugated BAs and unconjugated BAs in the ileum. Data are shown as the mean ± SEM (n = 6). *p < 0.05, **p < 0.01. ND: normal diet; LD: lithogenic diet; GLPH: high-dose *Ganoderma lucidum* polysaccharide; BA: bile acid

BAs act as endogenous signaling molecules that bind to FXR and modulate BA and cholesterol metabolism [12]. To determine the underlying mechanism by which GLP inhibits intestinal FXR signaling, we analyzed BA profiles in the distal ileum, where 95% of BAs are reabsorbed. The top 18 most abundant BA species and concentrations in the ileum were showed in Fig. 4A–C. The levels of total conjugated BAs and unconjugated BAs increased in the LD group, while GLP supplementation significantly reduced their levels (Fig. 4D). Notably, we found that the levels of CA, taurocholic acid (TCA), DCA and taurodeoxycholic acid (TDCA) were significantly elevated, but the β-MCA level was decreased in the ileum of LD-fed mice. Interestingly, GLP supplementation reversed these alterations (Fig. 4A, B). These findings suggested that GLP may alleviate LD-induced activation of intestinal FXR signaling by reducing FXR-agonistic BAs and increasing FXR-antagonistic BAs in ileum.

In addition, the total BA levels in liver and serum were also measured, showing that hepatic BA level was increased and serum BA level was reduced by GLP supplementation (Additional file 1: Fig. S6A, B).

### GLP reduced serum H_2_S level by improving microbiota dysbiosis

**Fig. 5:**
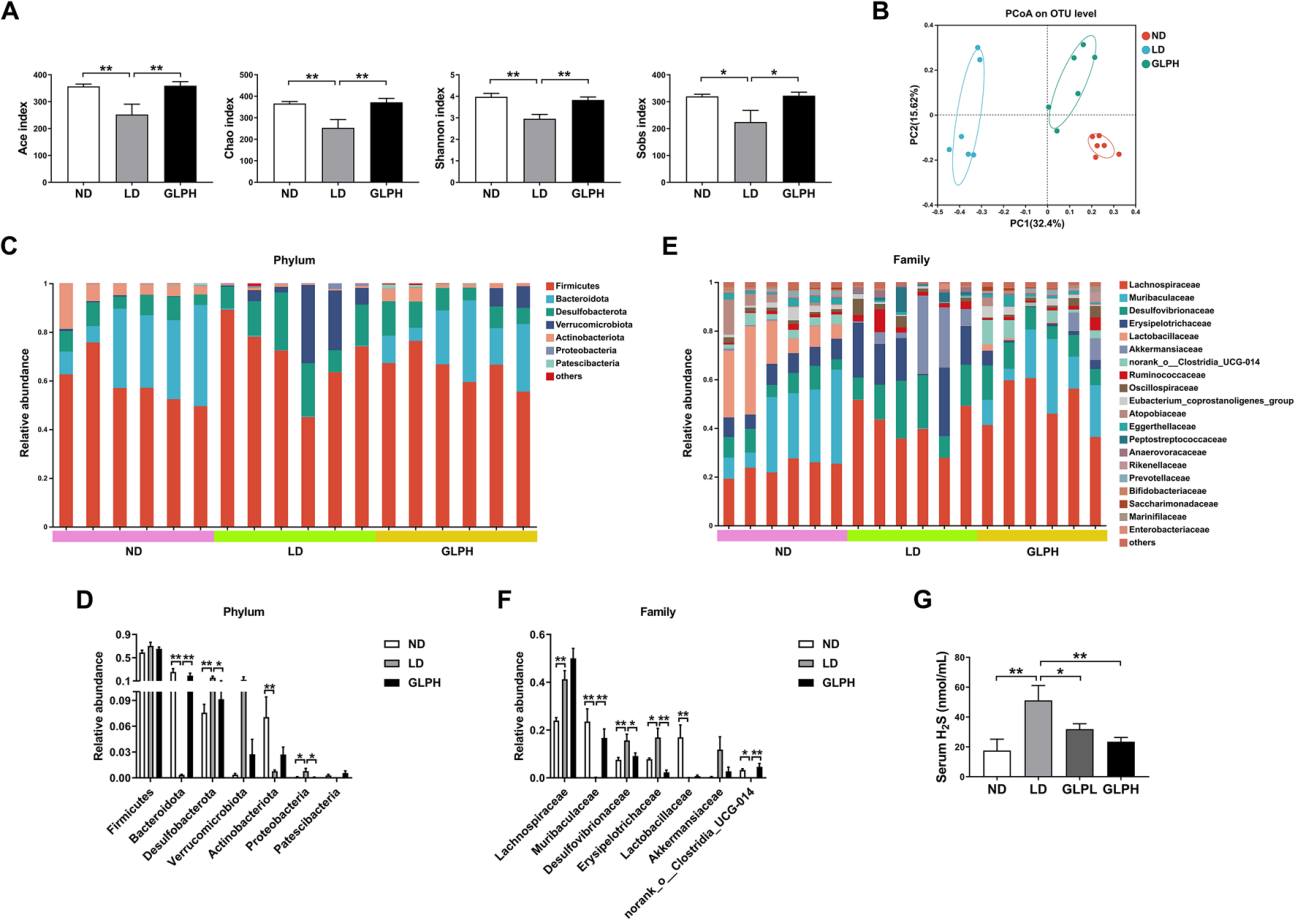
GLP modulated the composition of gut microbiota and reduced serum H_2_S level in LD-fed mice. **A** Ace, Chao, Shannon and Sobs index (n = 6). **B** PCoA analysis among groups (n = 6). **C** Relative abundances of phyla (n = 6). **D** Multigroup comparisons of the top 7 most abundant phyla (n = 6). **E** Relative abundances of families (n = 6). **F** Multigroup comparisons of the top 7 most abundant families (n = 6). **G** Serum H_2_S level (n = 10). Data are shown as the mean ± SEM. *p < 0.05, **p < 0.01. ND: normal diet; LD: lithogenic diet; GLPL: low-dose *Ganoderma lucidum* polysaccharide; GLPH: high-dose *Ganoderma lucidum* polysaccharide; PCoA: principal coordinate analysis; H_2_S: hydrogen sulfide

A previous study has demonstrated that *Desulfovibrionales*, along with its metabolic product H_2_S, can induce hepatic FXR and inhibit BA synthesis, thereby promoting CG formation [16]. In order to further explore the potential mechanism by which GLP inhibits hepatic FXR signaling, we analyzed the intestinal microbiota using 16S rRNA gene sequencing. Alpha diversity indices, including Ace, Chao, Shannon and Sobs, were used to describe species richness, diversity and evenness. As shown in Fig. 5A, these indices exhibited significant decreases in LD-fed mice, while GLP supplementation significantly increased these indices. PCoA revealed that microbiota structure of LD-fed mice was distinct from that of ND-fed mice, whereas GLP supplementation had a significant impact on the microbiota structure in LD-fed mice (Fig. 5B).

Next, we examined the gut microbiota structure at different levels. Figure 5C showed the top 7 most abundant phyla and relative abundances. LD-fed mice exhibited a lower abundance of *Bacteroidota* and higher abundances of *Desulfobacterota* and *Proteobacteria* compared to the ND group. However, GLP supplementation reversed these alterations (Fig. 5C, D). Previous studies have reported the beneficial impact of *Bacteroidota* on obesity [33], and the significant correlation between *Proteobacteria* and liver steatosis [34]. The top 20 most abundant families and relative abundances were showed in Fig. 5E. At the family level, the relative abundances of *Muribaculaceae* and *Lactobacillaceae*, which play important roles in ameliorating metabolic disorders [33], were significantly decreased in the LD group, however, GLP supplementation significantly increased the abundance of *Muribaculaceae*. Conversely, the LD group exhibited significant increases in the abundances of *Desulfovibrionaceae* and *Erysipelotrichaceae*, while GLP reduced their abundances (Fig. 5E, F). *Desulfovibrionaceae* and *Erysipelotrichaceae* have been reported to be associated with metabolic disorders and may be the potential causes of obesity [35]. Notably, the relative abundances of the order *Desulfovibrionales* and the family *Desulfovibrionaceae* were increased in the LD group, while GLP reversed the upregulation (Fig. 5E, F and Additional file 1: Fig. S7A, B). In addition, GLP significantly decreased serum H_2_S levels, which were increased by LD consumption (Fig. 5G).

These results indicated that GLP may alleviate LD-induced activation of hepatic FXR signaling by improving gut microbiota dysbiosis, particularly by decreasing the relative abundances of the order *Desulfovibrionales* and the family *Desulfovibrionaceae*, and reducing serum H_2_S levels.

### FXR activation attenuated the protective effects of GLP on CG formation

**Fig. 6:**
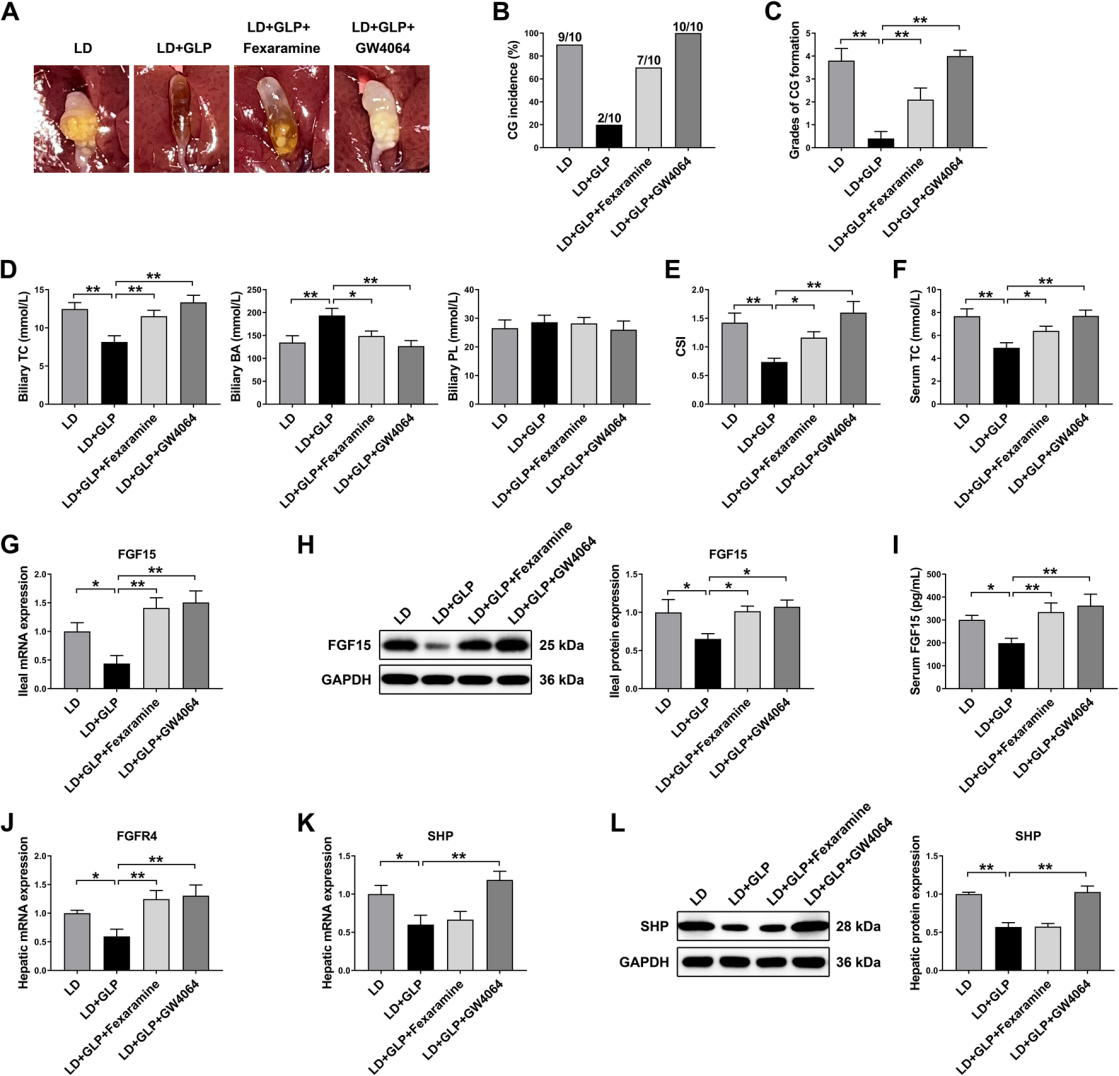
FXR activation reversed the protective effects of GLP in LD-fed mice. **A** Representative gallbladders and gallstones. **B** Incidence of gallstone. **C** Grades of experimental CGs (n = 10). **D** Concentrations of TC, BA and PL in gallbladder bile (n = 10). **E** CSI of gallbladder bile (n = 10). **F** Serum TC level (n = 10). **G** The mRNA expression of FGF15 in the ileum (n = 8). **H** The protein expression and quantification of FGF15 in the ileum (n = 6). **I** Serum FGF15 level (n = 10). **J**, **K** The mRNA expression of FGFR4 and SHP in the liver (n = 8). **L** The protein expression and quantification of SHP in the liver (n = 6). The mRNA expression and protein expression were normalized to GAPDH and shown as fold changes relative to control group. Data are shown as the mean ± SEM. *p < 0.05, **p < 0.01. LD: lithogenic diet; GLP: *Ganoderma lucidum* polysaccharide; CG: cholesterol gallstone; TC: total cholesterol; BA: bile acid; PL: phospholipid; CSI: cholesterol saturation index

The results presented above suggested that GLP may improve CG formation by inhibiting intestinal and hepatic FXR signaling in LD-fed mice. To further confirm the beneficial effects of FXR inhibition by GLP, we conducted a follow-up recovery experiment using LD-fed mice treated with GLP (4% in LD), and GLP coupled with 100 mg kg^−1^ d^−1^ fexaramine, a intestine-specific FXR agonist [36], and GW4064, a non-selective FXR agonist [36], for 6 weeks. Fexaramine and GW4064 markedly reversed the protective effect of GLP against CG formation (Fig. 6A). The incidence and grade of gallstones were significantly increased by fexaramine and GW4064, but the increase was more remarkable with GW4064 (Fig. 6B, C). There were no significant differences in body weight gain among groups (Additional file 1: Fig. S8A). Subsequently, we analyzed lipid composition in gallbladder bile and observed that both fexaramine and GW4064 significantly reversed the decrease in TC levels and the increase in total BA levels induced by GLP, resulting in an increase in the CSI of gallbladder bile (Fig. 6D, E and Additional file 1: Fig. S8B). Furthermore, GLP-induced reduction in serum TC level was significantly reversed by fexaramine and GW4064 (Fig. 6F). Importantly, the effects observed with GW4064 were more pronounced than those observed with fexaramine, suggesting that it was the global FXR inhibition that contributed to the improvement of CG formation by GLP, rather than solely intestinal FXR inhibition.

Furthermore, we investigated the impact of fexaramine and GW4064 on FXR signaling in the ileum and liver. As shown in Fig. 6G–I, both fexaramine and GW4064 significantly increased the mRNA and protein expression of FGF15 in ileum as well as the serum concentration of FGF15, which were reduced by GLP supplementation. Hepatic FGFR4 mRNA expression was upregulated in parallel with the increased ileal FGF15 mRNA and protein expression and serum FGF15 levels with fexaramine and GW4064 intervention (Fig. 6J). In addition, both the mRNA and protein data indicated that hepatic SHP expression was markedly upregulated by GW4064, but not by the intestine-specific FXR agonist fexaramine (Fig. 6K, L).

In summary, these findings suggested that GLP attenuated CG formation through an intestinal and hepatic FXR-dependent mechanism.

## Discussion

In the current study, we found that GLP supplementation effectively attenuated LD-induced CG formation in mice. GLP supplementation resulted in a reduction in the TC levels and an increase in the total BA levels, while also decreasing the CSI in gallbladder bile. Furthermore, GLP supplementation downregulated the FXR-FGF15 signaling pathway in the intestine and FXR-SHP signaling pathway in the liver, leading to enhanced hepatic BA synthesis and increased cholesterol depletion. Additionally, GLP supplementation reduced hepatic cholesterol synthesis and secretion. Mechanistically, GLP inhibited FXR signaling by (i) reducing the levels of FXR-agonistic BAs such as CA and DCA, while increasing the level of FXR-antagonistic BA (β-MCA) in the ileum; (ii) ameliorating LD-induced gut microbiota dysbiosis and reducing the serum level of H_2_S, a known hepatic FXR inducer [16].

The LD-induced mouse CG model has been widely utilized due to its simplicity, high success rate, good repeatability and easy observability. The LD is mainly characterized as high cholesterol levels, as excessive intake of cholesterol is a significant factor for CG formation. In 1964, Tepperman et al. [37] first discovered that incorporating a combination of cholesterol and CA in feed could effectively establish the mouse model of gallstones, suggesting the necessity of CA for gallstone formation. Since then, researchers have induced CG formation in mice by feeding them a high-fat, high-cholesterol diet with a certain proportion of CA [38, 39]. Based on previous studies, we employed a diet consisting of 15% fat, 1.25% cholesterol, 0.5% CA to induce a mouse CG model for investigating the impact of GLP on CG formation and exploring the potential mechanisms.

The occurrence of CGs is associated with metabolic diseases such as obesity, diabetes, non-alcoholic fatty liver disease (NAFLD), cardiovascular disease. There is a certain correlation between the development of CG diseases and metabolic disorders [40, 41]. For instance, the activation of hypoxia inducible factor 1α subunit (HIF1A) in steatotic liver contributed to CG formation [42]. In patients with diabetes, hepatic insulin resistance (IR) could promote CG formation by upregulating the expression of hepatic ABCG5 and ABCG8 [43]. Numerous studies have demonstrated that GLP can improve obesity, hyperlipidemia and hepatic steatosis [44, 45]. In addition, GLP has been shown to have hypoglycemic effects and can be used for the prevention and treatment of diabetes [46]. Our study found that GLP not only reduced the CG formation but also alleviated hepatic steatosis and dyslipidemia in LD-fed mice.

The abnormal metabolism of BAs and cholesterol is the main cause of CG formation [40]. FXR is well known for its role in regulating BA, carbohydrate and lipid metabolism [47]. Intestinal FXR-FGF15 and hepatic FXR-SHP signaling are the main negative regulators of BA synthesis and are critical for the maintenance of BA homeostasis [12]. When FXR is activated, it downregulates the expression of genes encoding BA synthetic enzymes in liver, thereby reducing BA synthesis. Our research found that GLP decreased the expression of FGF15 in ileum and SHP in liver, indicating that GLP could inhibit LD-induced activation of intestinal and hepatic FXR. Consequently, GLP increased the expression of CYP7A1 and CYP7B1 in liver, promoting BA synthesis from cholesterol and increasing cholesterol depletion. Hepatic hypersecretion of cholesterol is an important contributor to CG formation [40]. Overexpression of sterol efflux transporters ABCG5/G8 increases the biliary cholesterol level, thus increasing the risk of cholesterol crystal precipitation and CG formation [16, 41]. Studies have showed that variants of ABCG5/G8 are associated with CGs in various populations [48]. Our results showed that GLP decreased the expression of SREBP2, ABCG5 and ABCG8 in liver, suggesting that GLP can inhibit hepatic cholesterol synthesis and reduce the secretion of cholesterol into bile. Consistent with these findings, GLP also reduced the cholesterol levels in the liver and gallbladder bile. These results suggested that GLP can inhibit intestinal and hepatic FXR signaling, regulate BA and cholesterol metabolism, and attenuate LD-induced biliary cholesterol supersaturation.

As endogenous ligands of FXR, BAs play crucial roles in regulating various physiological processes in the host [49]. Most BAs are reabsorbed in the terminal ileum. Dietary fibers have been shown to interact with BAs in the intestine. This interaction may partially prevent BAs from being reabsorbed into the enterohepatic circulation, resulting in an increase in BA excretion [50]. In our study, we observed that the levels of CA and DCA were increased in the terminal ileum of LD-fed mice. This increase may be attributed to the high concentration of CA in the feed, which can be converted into DCA by intestinal bacteria. However, GLP supplementation resulted in decreased levels of CA and DCA in the ileum. This decrease may be due to the interactions between GLP and BAs in the intestine, leading to reduced reabsorption of BAs. The decrease of intestinal BA reabsorption is considered to be the main mechanism underlying the cholesterol-lowering effect of dietary fiber [51]. In vivo studies showed that ingestion or administration of dietary fibers could increase the excretion of BAs in feces [52], leading to a reduction in cholesterol by diversion to BA synthesis [53–55]. Our results showed that GLP upregulated the expression of CYP7A1 and CYP7B1 in the liver to promote BA synthesis, which may be due to increased BA excretion in feces. Consistent with the enhanced hepatic BA synthesis and reduced intestinal BA reabsorption, our data showed that GLP supplementation significantly increased hepatic levels and reduced serum levels of total BAs. In addition, we also found that GLP could increase the level of β-MCA in the ileum. As reported, β-MCA can prevent the formation of experimental CGs in mice [56]. These results revealed that GLP reduced the levels of FXR-agonistic BAs and increased the levels of FXR-antagonistic BAs in the ileum, which may contribute to the inhibition of intestinal FXR signaling upon GLP supplementation.

There is a potential link between gut microbiota dysbiosis and the formation of CGs [57, 58]. Previous research has shown that alteration of indigenous gastrointestinal microbiota through bacteria transferring can induce CG formation in germ-free mice [57]. Studies have revealed that GLP can improve the dysbiosis of gut microbiota [18, 20]. In our study, we observed that GLP supplementation alleviated the reduced-richness and diversity of intestinal microbiota and modulated the gut microbiota structure at different levels in LD-fed mice. Specifically, GLP decreased the abundances of bacteria that are detrimental to metabolism, such as *Proteobacteria* at the phylum level, *Desulfovibrionales* at the order level, and *Desulfovibrionaceae* and *Erysipelotrichaceae* at the family level. Conversely, GLP increased the abundances of bacteria that are beneficial to metabolism, such as the phylum *Bacteroidota* and the family *Muribaculaceae*. It is worth noting that the order *Desulfovibrionales* and the family *Desulfovibrionaceae* are types of bacteria that can reduce sulfate to H_2_S [59], and produce endotoxins [60]. H_2_S can damage intestinal mucosa and disrupt intestinal barrier function, which may contribute to the development of intestinal inflammation and cancer [35]. Endotoxin can trigger an immune response upon entering the host circulatory system, which may be the potential etiology of chronic diseases induced by high-fat diet (HFD) [35]. A recent study [16] found that patients with CGs had an overabundance of *Desulfovibrionales*, and introducing *Desulfovibrionales* to gallstone-resistance mice could lead to gallstone formation. The increased abundance of *Desulfovibrionales* in the gut microbiota resulted in the production of H_2_S, which could activate hepatic FXR and suppress the expression of CYP7A1 and BA synthesis. This mechanism is believed to contribute to gallstone formation [16]. Our study showed that LD-fed mice had higher levels of *Desulfovibrionales* and serum H_2_S, but these changes were reversed upon GLP supplementation. These results suggested that GLP can alleviate the dysbiosis caused by LD and drive a shift in gut microbiota composition towards a healthy condition. In addition, GLP also reduced serum H_2_S levels, which may be responsible for the inhibition of hepatic FXR signaling upon GLP supplementation.

The recovery study using FXR agonists further confirmed the important role of FXR signaling in the protective effect of GLP against CG formation. In our study, both the global FXR agonist GW4064 and the gut-restricted FXR agonist fexaramine markedly reversed the protective effect of GLP, leading to increased biliary CSI and promoting gallstone formation. In addition, we also found that both GW4064 and fexaramine counteracted the lowering effect of GLP on serum cholesterol. Notably, the reversal effect was more pronounced with GW4064 intervention compared to fexaramine. These findings demonstrated that both intestinal and hepatic FXR signaling are crucial for the protective effect of GLP against CG formation.

Considering that ABCG5/G8 expression can be regulated not only by FXR [61, 62] but also by liver X receptor α (LXRα) [63, 64], whether GLP ameliorates CG formation by regulating additional FXR-independent pathways is still unclear. In our study, the protective actions of GLP are primarily attributable to its regulation of the FXR signaling pathway. Although our findings suggest a new strategy for the prevention against CG formation, there are differences in BA composition between humans and mice [65] and additional research is needed before translation into clinical practice.

## Conclusions

In conclusion, our study demonstrated that GLP effectively inhibited CG formation by suppressing intestinal and hepatic FXR signaling. The decrease in ileal CA, DCA, the increase in ileal β-MCA, the decrease in the abundance of *Desulfovibrionales* and concomitant decrease in serum H_2_S may be responsible for the inhibition of FXR by GLP, leading to increased hepatic BA synthesis. Additionally, GLP inhibited hepatic cholesterol synthesis and secretion. These alterations in BA and cholesterol metabolism resulted in a reduction in cholesterol supersaturation of gallbladder bile and a lower incidence of CGs by GLP. Based on these findings, GLP shows promise as a potential preventive strategy for CG disease.

### Supplementary Information

**Additional file 1: Fig. S1.** Characterization of molecular weight of GLP. **Fig. S2.** Characterization of monosaccharide composition of GLP. **Fig. S3.** Characterization of functional groups of GLP. **Fig. S4.** The body weight and biliary lipid composition of mice in the GLP intervention study. **Fig. S5.** The effect of GLP on biliary phospholipid secretion. **Fig. S6.** GLP altered the total BA levels in liver and serum. **Fig. S7.** GLP modulated gut microbiota at the order level. **Fig. S8**: The body weight and biliary lipid composition of mice in the FXR regulation study.

## Data Availability

All data are available in the main text or the Supplementary Materials. The sequencing raw data are available from the corresponding author upon request.

## Abbreviations

ABCB4: ATP-binding cassette subfamily B member 4
ABCG5: ATP-binding cassette subfamily G member 5
ABCG8: ATP-binding cassette subfamily G member 8
ANOVA: Analysis of variance
Ara: Arabinose
ATR-FTIR: Attenuated total reflectance Fourier transform infrared
BA: Bile acid
BSEP: Bile salt export pump
CA: Cholic acid
CG: Cholesterol gallstone
CSI: Cholesterol saturation index
CYP7A1: Cholesterol 7α-hydroxylase
CYP27A1: Sterol 27-hydroxylase
CYP7B1: Oxysterol 7α-hydroxylase
CYP8B1: Sterol 12α-hydroxylase
DCA: Deoxycholic acid
ECL: Enhanced chemiluminescence
FGF15: Fibroblast growth factor 15
FGFR4: Fibroblast growth factor receptor 4
Fuc: Fucose
FXR: Farnesoid X receptor
Gal: Galactose
GalA: Galacturonic acid
GalN: Galactosamine
GalNAc: *N*-Acetylgalactosamine
GAPDH: Glyceraldehyde 3-phosphate dehydrogenase
Glc: Glucose
GlcA: Glucuronic acid
GlcN: Glucosamine
GlcNAc: *N*-Acetylglucosamine
GLP: *Ganoderma lucidum* Polysaccharide
GulA: Guluronic acid
H&E: Hematoxylin and eosin
HIF1A: Hypoxia inducible factor 1α subunit
HPGPC: High-performance gel permeation chromatography
HPLC: High-performance liquid chromatography
H_2_S: Hydrogen sulfide
IR: Insulin resistance
LC–MS/MS: Liquid chromatography-tandem mass spectrometry
LD: Lithogenic diet
LXRα: Liver X receptor α
Man: Mannose
ManA: Mannuronic acid
β-MCA: β-Muricholic acid
MRP2: Multidrug resistance-associated protein 2
MS: Metabolic syndrome
NAFLD: Non-alcoholic fatty liver disease
OUT: Operational taxonomic unit
PCoA: Principal coordinate analysis
PL: Phospholipid
QIME: Quantitative Insights into Microbial Ecology
RDP: Ribosomal Database Project
Rha: Rhamnose
Rib: Ribose
RT-qPCR: Real-time quantitative polymerase chain reaction
SCAP: SREBP cleavage-activating protein
SEM: Standard error of the mean
SHP: Small heterodimer partner
SREBP2: Sterol regulatory element-binding protein 2
16S rRNA: 16S ribosomal RNA
TC: Total cholesterol
TCA: Taurocholic acid
TDCA: Taurodeoxycholic acid
TG: Triglyceride
UDCA: Ursodeoxycholic acid
UPLC: Ultraperformance liquid chromatography
Xyl: Xylose
