# Supplementary material for: Ganoderma lucidum polysaccharide ameliorates cholesterol gallstone formation by modulating cholesterol and bile acid metabolism in an FXR-dependent manner
Source: Chin Med. 2024 Jan 24;19:16. doi: 10.1186/s13020-024-00889-y (PMC10809463; doi:10.1186/s13020-024-00889-y)
Supplement: Supplementary file 1 — Additional file 1: Fig. S1. Characterization of molecular weight of GLP. Fig. S2. Characterization of monosaccharide composition of GLP. Fig. S3. Characterization of functional groups of GLP. Fig. S4. The body weight and biliary lipid composition of mice in the GLP intervention study. Fig. S5. The effect of GLP on biliary phospholipid secretion. Fig. S6. GLP altered the total BA levels in liver and serum. Fig. S7. GLP modulated gut microbiota at the order level. Fig. S8: The body weight and biliary lipid composition of mice in the FXR regulation study. [file 13020_2024_889_MOESM1_ESM.docx]

**Additional file**

**Additional figures and figure legends**

***Ganoderma lucidum* polysaccharide ameliorates cholesterol gallstone formation by modulating cholesterol and bile acid metabolism in an FXR-dependent manner**

Dan Huang^1†^, Shuang Shen^1†^, Qian Zhuang^1†^, Xin Ye^1^, Yueqin Qian^1^, Zhixia Dong^1*^ and Xinjian Wan^1*^

^1^Digestive Endoscopic Center, Shanghai Sixth People's Hospital Affiliated to Shanghai Jiao Tong University School of Medicine, Shanghai, China

^†^Dan Huang, Shuang Shen and Qian Zhuang have contributed equally to this work.

***Correspondence:**

Xinjian Wan

E-mail: slwanxinjian2020@126.com

Mailing address: Digestive Endoscopic Center, Shanghai Sixth People's Hospital Affiliated to Shanghai Jiao Tong University School of Medicine, No. 600 Yishan Road, Shanghai 200233, China

Zhixia Dong

E-mail: dzhixia2013@163.com

Mailing address: Digestive Endoscopic Center, Shanghai Sixth People's Hospital Affiliated to Shanghai Jiao Tong University School of Medicine, No. 600 Yishan Road, Shanghai 200233, China


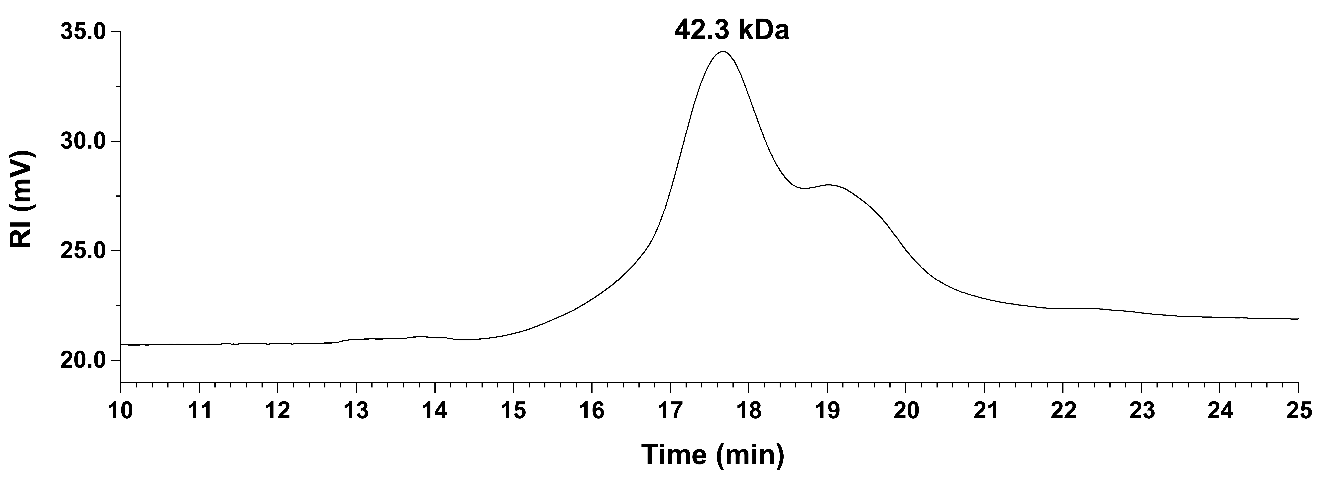
**Fig. S1. Characterization of molecular weight of GLP.** The molecular weight of GLP as determined by HPGPC analysis. GLP, *Ganoderma lucidum* polysaccharide; HPGPC, high-performance gel permeation chromatography.


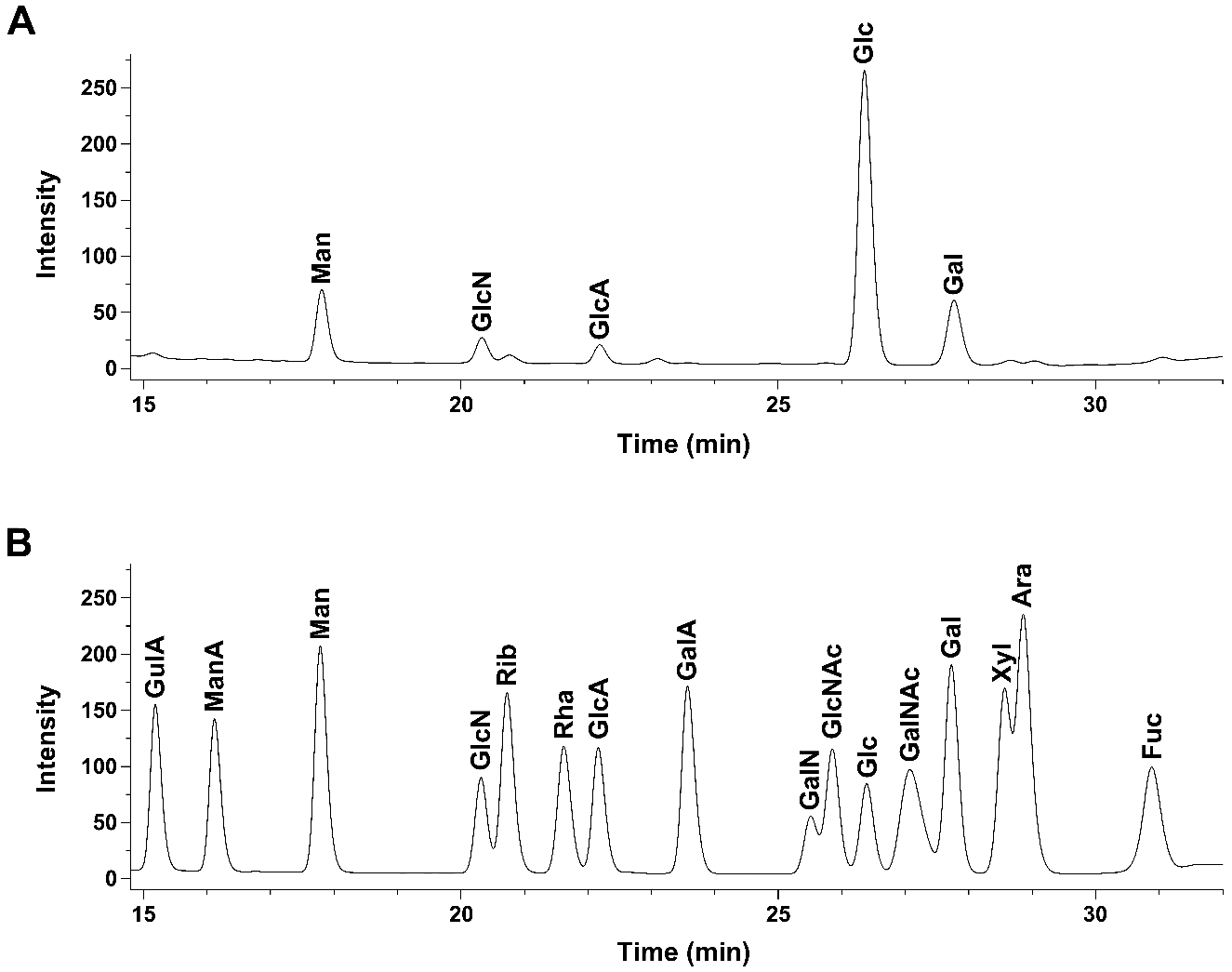
**Fig. S2. Characterization of monosaccharide composition of GLP.** The monosaccharide composition of GLP (**A**) and standard monosaccharides (**B**) as determined by HPLC analysis. GulA, guluronic acid; ManA, mannuronic acid; Man, mannose; GlcN, glucosamine; Rib, ribose; Rha, rhamnose; GlcA, glucuronic acid; GalA, galacturonic acid; GalN, galactosamine; GlcNAc, N-acetylglucosamine; Glc, glucose; GalNAc, N-acetylgalactosamine; Gal, galactose; Xyl, xylose; Ara, arabinose; Fuc, fucose; GLP, *Ganoderma lucidum* polysaccharide; HPLC, high-performance liquid chromatography.


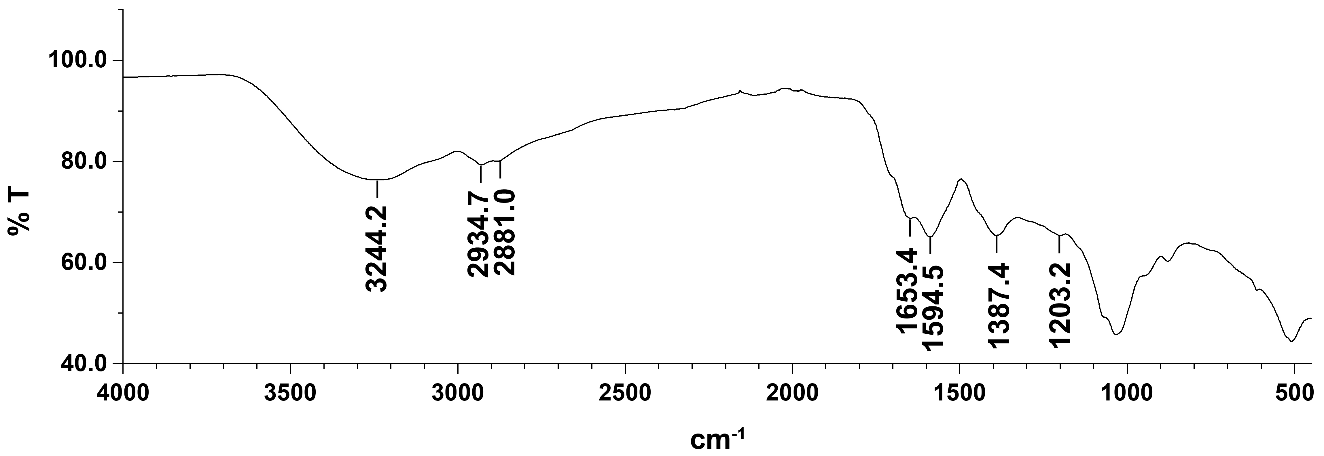
**Fig. S3. Characterization of functional groups of GLP.** The functional groups of GLP as determined by ATR-FTIR analysis. GLP, *Ganoderma lucidum* polysaccharide; ATR-FTIR, attenuated total reflectance Fourier transform infrared.

**
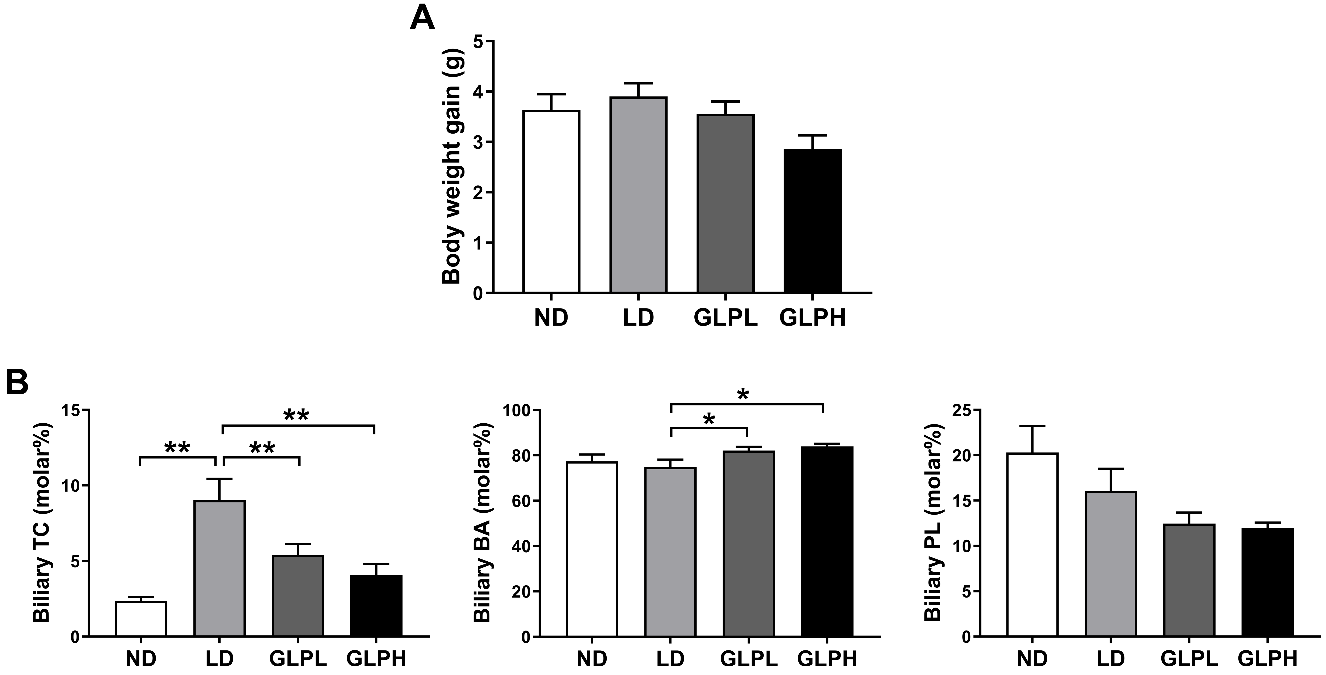
Fig. S4. The body weight and biliary lipid composition of mice in the GLP intervention study.** (**A**) The body weight gain. (**B**) Molar percentages of TC, BA and PL in gallbladder bile. Data are shown as the mean ± SEM (n = 10). *p < 0.05, **p < 0.01. ND, normal diet; LD, lithogenic diet; GLPL, low-dose *Ganoderma lucidum* polysaccharide; GLPH, high-dose *Ganoderma lucidum* polysaccharide; TC, total cholesterol; BA, bile acid; PL, phospholipid.


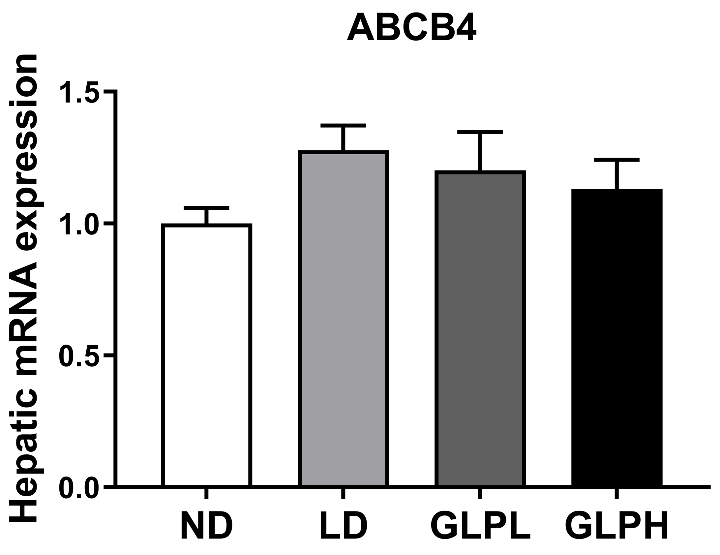
**Fig. S5. The effect of GLP on biliary phospholipid secretion.** The mRNA expression of ABCB4 in the liver. The mRNA expression was normalized to GAPDH and shown as fold changes relative to control group. Data are shown as the mean ± SEM (n = 8). ND, normal diet; LD, lithogenic diet; GLPL, low-dose *Ganoderma lucidum* polysaccharide; GLPH, high-dose *Ganoderma lucidum* polysaccharide.


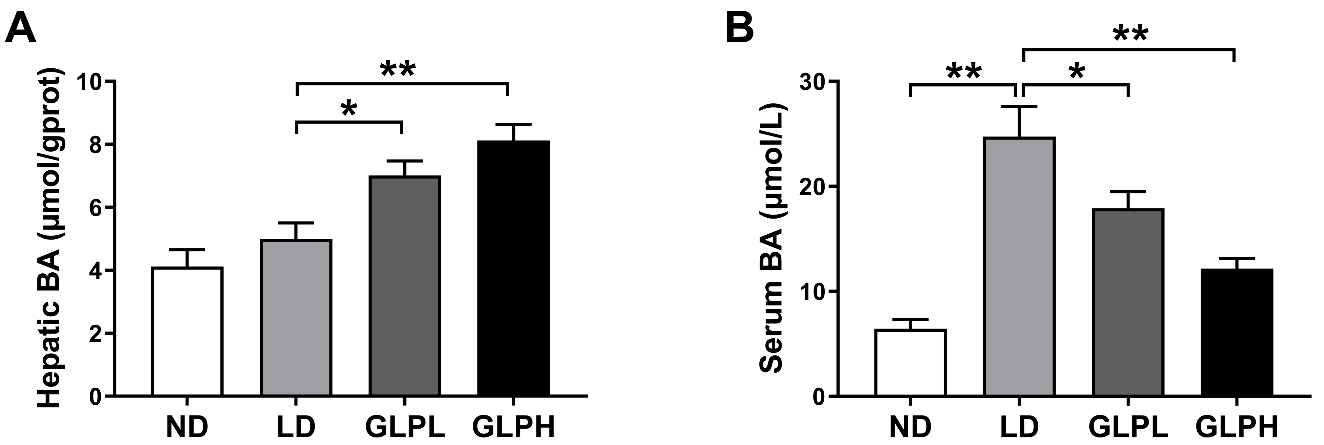
**Fig. S6. GLP altered the total BA levels in liver and serum.** (**A**) Hepatic levels of total BAs. (**B**) Serum levels of total BAs. Data are shown as the mean ± SEM (n = 10). *p < 0.05, **p < 0.01. ND, normal diet; LD, lithogenic diet; GLPL, low-dose *Ganoderma lucidum* polysaccharide; GLPH, high-dose *Ganoderma lucidum* polysaccharide; BA, bile acid.


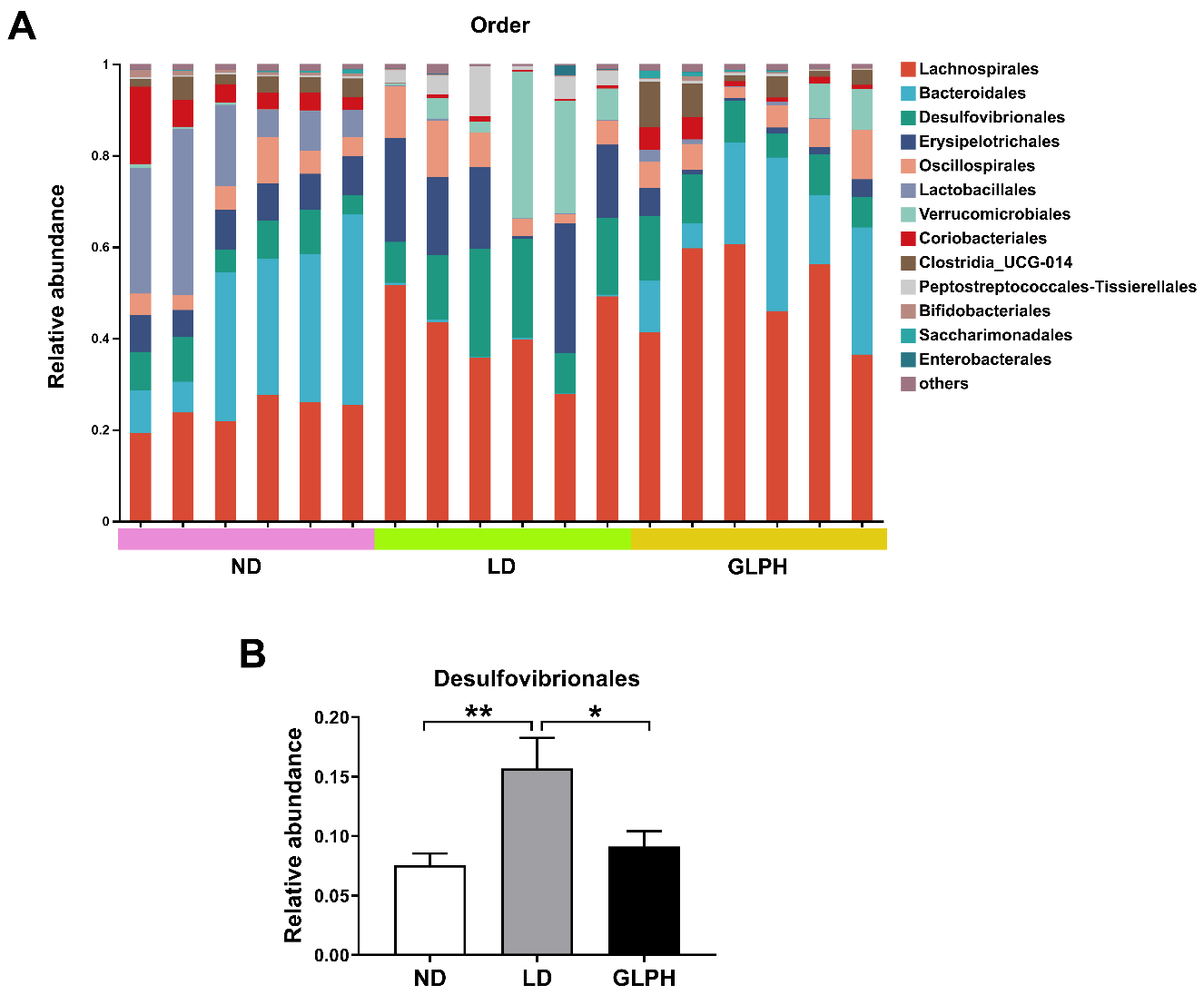
**Fig. S7. GLP modulated gut microbiota at the order level.** (**A**) Relative abundances of orders. (**B**) Comparison of the order *Desulfovibrionales*. Data are shown as the mean ± SEM (n = 6). *p < 0.05, **p < 0.01. ND, normal diet; LD, lithogenic diet; GLPH, high-dose *Ganoderma lucidum* polysaccharide.


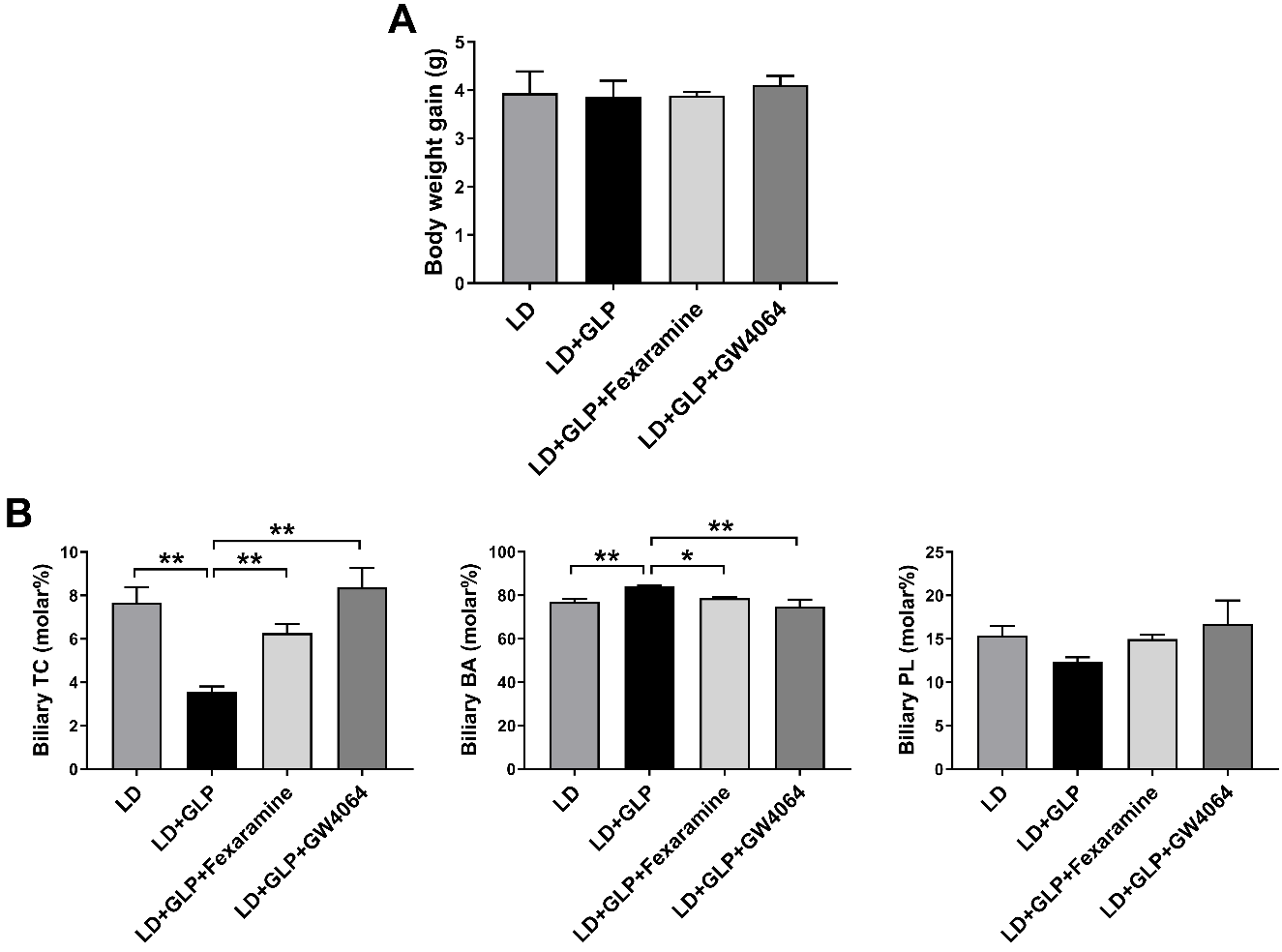
**Fig. S8. The body weight and biliary lipid composition of mice in the FXR regulation study.** (**A**) The body weight gain. (**B**) Molar percentages of TC, BA and PL in gallbladder bile. Data are shown as the mean ± SEM (n = 10). *p < 0.05, **p < 0.01. LD, lithogenic diet; GLP, *Ganoderma lucidum* polysaccharide; TC, total cholesterol; BA, bile acid; PL, phospholipid.
